# Spreading out Muscle Mass within a Hill-Type Model: A Computer Simulation Study

**DOI:** 10.1155/2012/848630

**Published:** 2012-11-22

**Authors:** Michael Günther, Oliver Röhrle, Daniel F. B. Haeufle, Syn Schmitt

**Affiliations:** ^1^Institut für Sport-und Bewegungswissenschaft, Universität Stuttgart, Allmandring 28, 70569 Stuttgart, Germany; ^2^Lehrstuhl für Bewegungswissenschaft, Institut für Sportwissenschaft, Friedrich-Schiller-Universität, Seidelstraße 20, 07749 Jena, Germany; ^3^Stuttgart Research Centre for Simulation Technology, Pfaffenwaldring 7a, 70569 Stuttgart, Germany; ^4^Institut für Mechanik (Bauwesen), Universität Stuttgart, Lehrstuhl II, Pfaffenwaldring 7a, 70569 Stuttgart, Germany

## Abstract

It is state of the art that muscle contraction dynamics is adequately described by a hyperbolic relation between muscle force and contraction velocity (Hill relation), thereby neglecting muscle internal mass inertia (first-order dynamics). Accordingly, the vast majority of modelling approaches also neglect muscle internal inertia. Assuming that such first-order contraction dynamics yet interacts with muscle internal mass distribution, this study investigates two questions: (i) what is the time scale on which the muscle responds to a force step? (ii) How does this response scale with muscle design parameters? Thereto, we simulated accelerated contractions of alternating sequences of Hill-type contractile elements and point masses. We found that in a typical small muscle the force levels off after about 0.2 ms, contraction velocity after about 0.5 ms. In an upscaled version representing bigger mammals' muscles, the force levels off after about 20 ms, and the theoretically expected maximum contraction velocity is not reached. We conclude (i) that it may be indispensable to introduce second-order contributions into muscle models to understand high-frequency muscle responses, particularly in bigger muscles. Additionally, (ii) constructing more elaborate measuring devices seems to be worthwhile to distinguish viscoelastic and inertia properties in rapid contractile responses of muscles.

## 1. Introduction

In case the force-velocity relation of a muscle (usually hyperbolic [1]: the Hill relation) is interpreted as a force law and coupled to inertia loads as, for example, in computer models of musculoskeletal multibody systems, it adds first-order dynamics to the systems' mechanical equations of motion. Usually, the force-velocity relations of a specific isolated muscle preparation (according to [2–4]: earliest known experiments by Jan Swammerdam around 1663; later: e.g., [1, 5–19]) or lumped-muscle assemblies (e.g., [20–22]) are determined through isotonic (e.g., [10, 13, 18]) or isokinetic (e.g., [7, 10, 16, 17]) contractions or by accelerating external inertia loads (e.g., [20, 21]).

In the isokinetic condition, a potential external inertia load would be nonaccelerated, whereas the muscle internal velocity distribution could not yet be guaranteed to have cancelled out (maybe internally and locally accelerated). Hypothesising that the muscle consists internally of just two parts arranged in series, the contractile element (CE) and the serial (visco-)elastic element (SEE), the isotonic condition is often chosen to separate an assumed subsequent steady-state response of the CE (and, thus, its isolated properties) from the so-called “initial elastic response” of a muscle. The latter is the finite period (in fibres below a millisecond [13, 23–27]) after a quick release in which the force settles to a new level, as mostly presumed, through mere SEE properties. In analogy to the isokinetic condition, however, the muscle internal force distribution cannot be guaranteed to have cancelled out during an isotonic contraction. That is, both contraction modes are prone to represent in fact nonsteady situations due to potential inertia loads involved, may they come from inertia of the measurement device in series or muscle internal masses themselves.

Some studies [28–30] have argued that muscle internal inertia forces can be neglected. All these authors based their argument on assuming that the force within one sarcomere accelerates just one other sarcomere, that is, a very low mass. In real muscle, however, all sarcomeres in parallel (imagine a cut through a muscle's cross-section) would have to accelerate all masses in series on both sides of the cut. Definitely, force generation of a real muscle cannot work without almost synchronous contraction along its whole mass distribution. Certainly, the force generated by one sarcomere will accelerate the inertia of more than its own and its direct neighbours. And indeed, there are some experimental studies [24–26, 31] in which high-frequency oscillatory responses to step excitations had been found, indicating that muscle inertia may interfere with contraction.

As a consequence of this and the fact that finite contraction distances have to be allowed during any contraction experiment, the measured results of both dynamic contraction conditions may delicately depend on muscle internal mass distribution. In fact, all mentioned experimental conditions are meant to finally determine the characteristics of the CE within the muscle. We can conclude that, at least theoretically, the so determined contractile muscle characteristic (force-velocity relation) might not be identified without taking inertia effects into account. Moreover, isolated preparations have been examined solely for invertebrate, arthropod, frog, toad, or small mammal muscles so far, that is, for muscle masses in the range 0.0001 ⋯ 10 g (maximum: 20 g in a turkey muscle [32]), thus, much smaller than those of humans or big mammals. Assuming geometric scaling, muscle force would just scale quadratically (cross-sectional area) with body length, whereas muscle mass would scale cubically (volume). Thus, accelerating force per muscle mass should decrease about linearly with body length. Accordingly, it may well be that first-order dynamics does not generally suffice to adequately describe muscle contraction dynamics. Therefore, in our view, at least two questions remain open. (i) Usually, experiments are performed on small muscles, whereas computer models often refer to upscaled muscles. Hence, the first question worth asking seems to be whether and how a mass distribution, scaled up to a design resembling bigger humans' or mammals' muscles, would bias the force-velocity relation. (ii) The second question is in regard to all muscle sizes. What is the time scale on which such inertia forces occur, especially compared to the time scale of the “initial elastic response”?

To answer these questions, we conducted a computer experiment in which modelling muscle dynamics was purposely reduced to the force-length and force-velocity dependencies of the CE. That is, we neglected any elasticity, whether in parallel or in series, to be able to focus solely on the CE force relationships. Usually, parallel elasticity does not intervene when starting the contraction just below or at about optimal fibre length. Serial elasticity can be neglected in case the contraction does not start in some pre-stretched condition like in, for example, activated isometry. Consequently, we calculated in this study how a muscle, which is modelled on the basis of classical Hill-type CE contraction dynamics, would be biased by and interact with muscle internal second-order dynamics (mass inertia). Thereby, the muscle is assumed to be fully activated initially and subjected to the maximum initial load gradient possible along its length, while being not prestretched: one muscle end is fixed, whereas the other end is released to contract freely.

## 2. Material and Methods

We simulated linear (onedimensional) dynamic muscle contractions using in-house software “simsys” [33–35] which incorporates the solver “de” [36] for time integration. The solver's absolute and relative error tolerances were set to 10^−12^.

**Figure 1:**
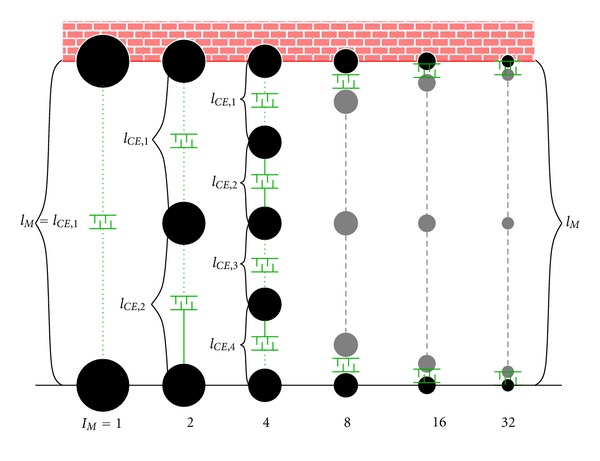
The six models used for simulating accelerated contractions of a muscle with an overall mass of 6.5 g are arranged from the left to the right. The models differ (i) by having split the muscle mass into an increasing number (*I*_*M*_ + 1) of point masses (PMs; depicted by circles) connected through a corresponding number *I*_*M*_ of CEs (depicted by “brushes”) and (ii) by distributing the optimal muscle length *l*_*M*,opt_ homogeneously to the optimal CE lengths *l*_CE,opt,*i*_ = *l*_*M*,opt_/*I*_*M*_. The sum of the circle areas, symbolising the muscle mass, is constant across the six models. The uppermost PM is always fixed to the world, whereas all other PMs can be accelerated by their adjacent CEs. Note that there is no gravity. Generally, *l*_*M*_ = ∑_*i*=1_^*I*_*M*_^ *l*_CE,*i*_ is the overall muscle length *l*_*M*_.

We approximated the continuous mass distribution along the muscle by modelling (Figure 1) a finite number *I*_*M*_ + 1 of discrete point masses (PMs) in an alternating sequence with a corresponding number of *I*_*M*_ contractile elements (CEs) indicated by “*i*”. To keep the model as simple as possible, we further assumed a homogeneous mass distribution, that is, the same 1/(*I*_*M*_ + 1) portion of the overall muscle mass *ℳ* is attributed to each PM. For all models with *I*_*M*_ = 1,2, 4,8, 16,32 CEs, we examined just one dynamic load situation: a fully active muscle (all activities *q*_*i*_ = 1, *q*_*i*_ ∈ [0,1]) contracting concentrically with one muscle end fixed to the world, and the other end is released to contract freely, ignoring gravity. This is the most dynamic, however, non-prestretched situation conceivable, because we chose all *I*_*M*_ CEs to be at their (common) optimal length (*l*_CE,*i*_ = *l*_CE,opt,*i*_ = *l*_CE,opt_) initially, not shortening (${\dot{l}}_{\text{CE},i}=0$; the dot “ ^·^” denotes the time *t* derivative) and, therefore, initially pulling with their (common) maximum isometric force (*F*_CE,*i*_ = *F*_max⁡,*i*_ = *F*_max⁡_). Consequently, the only PM to be accelerated initially was the one at the free end, being exposed to the maximum force gradient possible: *F*_CE,*I*_*M*__ = *F*_max⁡_ pulling on the one side, whereas there is no force counteracting on the other side. That is, we computed the decay of this singular force step while going through an intermittent scenario of a force (and acceleration) gradient distributed along the whole muscle length *l*_*M*_ = ∑_*i*=1_^*I*_*M*_^ *l*_CE,*i*_. With this decay, we quantified the time scale on which the muscle attunes to any load difference between its two ends, depending on its CE characteristics and internal inertia.

For this loading situation, we consider only two different muscle designs. Basic muscle model parameters are the muscle mass *ℳ*, the maximum isometric force *F*_max⁡_ of all the CEs and the overall muscle, and the optimal length *l*_CE,opt_ of all CEs determining the optimal over-all muscle length *l*_*M*,opt_ = ∑_*i*=1_^*I*_*M*_^ *l*_CE,opt,*i*_ = *I*_*M*_ · *l*_CE,opt_. The first muscle represents an averaged assembly of the four plantar flexors of a piglet [21]: *ℳ* = 6.5 g, *F*_max⁡_ = 30 N, *l*_*M*,opt_ = 0.015 m, the latter two parameters characterising static (isometric) muscle properties. Dynamic concentric contraction properties are characterised by two further parameters *A*_*rel*⁡_, *B*_*rel*⁡_ (see next paragraph), fixing the maximum concentric contraction velocity *v*_*M*,max⁡_ = 0.15 m/s and the curvature of an assumed hyperbolic force-velocity relation [1]. The second muscle is a scaled version of this piglet muscle with hundredfold mass (*ℳ* = 650 g). This comes from scaling the piglet's length (to *l*_*M*,opt_ = 0.15 m, *v*_*M*,max⁡_ = 1.5 m/s) as well as the cross-sectional area (meaning *F*_max⁡_ = 300 N), each by a factor of ten. Thus, both basic contraction parameters consistently scale tenfold, whereas the values of the dynamic parameters *A*_*rel*⁡_, *B*_*rel*⁡_ are retained unchanged. The human pectineus muscle [37] or the medial head of the horse triceps muscle [38] would be examples of such a muscle design.

Any muscle elasticity (parallel and in series to the CE) is neglected. Also, activation dynamics *q*_*i*_(*t*) is not taken into account (*q*_*i*_ = 1 during complete contractions). Concentric contraction dynamics of the CEs as applied in this study has been completely described elsewhere [21, 39]. Here, we give a short summary. The isometric force function of a CE is modelled as *F*_CE,isom,*i*_(*l*_CE,*i*_, *q*_*i*_) = *F*_max⁡_ · *q*_*i*_ · *f*_isom_(*l*_CE,*i*_), where *f*_isom_(*l*_CE,*i*_) ∈ [0,1] is constructed from two exponential (bell) curve branches (ascending and descending limb; see parameter values in [21]) steadily and differentiably connected at *l*_CE,opt,*i*_. Besides the isometric force parameter *F*_max⁡_, the CE's hyperbolic (Hill-type) force-velocity relation is determined by two further normalised (Hill) parameters *A*_*rel*⁡,*i*_ = *A*_*i*_/*F*_CE,isom,*i*_, *B*_*rel*⁡,*i*_ = *B*_*i*_/*l*_CE,opt,*i*_. *A*_*i*_, *B*_*i*_ are the hyperbola's asymptotes in nonnormalised units. They are generally, just like *F*_CE,isom,*i*_, rather functions of the CE state variables *l*_CE,*i*_, *q*_*i*_ than constant parameters [21, 40]. Together, both asymptotes fix *v*_max⁡,*i*_ and the curvature of the force-velocity relation. Our restriction to identical CEs which contract fully activated around optimal lengths is achieved by globally choosing *A*_*rel*⁡,*i*_ = *A*_*rel*⁡_ = 0.1 and *B*_*rel*⁡,*i*_ = *B*_*rel*⁡_ = 1.0 (1/s) [21] as constant parameters for each CE. Thus, *v*_max⁡,*i*_ = *v*_max⁡_ = *B*_*rel*⁡_/*A*_*rel*⁡_ · *l*_CE,opt_ is also (almost: see Section 3) a fixed parameter in our simulations. Therewith, *v*_*M*,max⁡_ = *I*_*M*_ · *v*_max⁡_ scales directly with *l*_*M*,opt_ in our model.

Generally, the change $dF\left(x,t\right)=\ddot{x}\left(x,t\right)\cdot dℳ$ in the force *F*(*x*, *t*) acting along the muscle longitudinal axis in *x*-direction runs in proportion to the mass portion *dℳ* located between position *x* and *x* + *dx* and to the respective current acceleration $\ddot{x}\left(x,t\right)$ of this mass portion. Note that *F*(*x*, *t*) means the force acting on *one side* of each mass portion, that is, represents the force in the corresponding adjacent CE. Our assumption of a homogeneous mass distribution (i.e., *ℳ*/*l*_*M*_ = const) is equivalent to a constant mass increment *dℳ* = *ℳ*/*l*_*M*_ · *dx* per change in position *dx*. Thus, in our model we find

(1) $$\begin{array}{c} dF\left(x,t\right)=\ddot{x}\left(x,t\right)\cdot ℳ/l_{M}\left(t\right)\cdot dx. \end{array}$$

The situation, in which the muscle is fixed at *x* = 0 and left free at *x* = *l*_*M*_, is characterised by

(2) $$\begin{array}{c} \ddot{x}\left(0,t\right)=0,\\ \ddot{x}\left(x_{\text{COM}},t\right)=\frac{F\left(0,t\right)-F\left(l_{M},t\right)}{ℳ}=\frac{{F|}_{x=0}\left(t\right)}{ℳ}, \end{array}$$

with the boundary conditions *F*(*l*_*M*_, *t*) = 0 and *F*(0, *t*) = *F*|_*x*=0_(*t*) applying at any time *t*. Now, if local accelerations were distributed as homogeneously as mass (implying *x*_COM_(*t*) = *l*_*M*_(*t*)/2) along the muscle, that is,

(3) $$\begin{array}{c} \ddot{x}\left(x,t\right)=a+b\cdot x, \end{array}$$

then, from comparing (3) with the two equations (2), we could deduce *a* = 0 and *b* = 2*F*|_*x*=0_(*t*)/(*ℳ* · *l*_*M*_(*t*)). For homogeneously distributed accelerations we, thus, find

(4) $$\begin{array}{c} {\ddot{x}|}_{x=l_{M}}\left(t\right)=\ddot{x}\left(l_{M},t\right)=\frac{2{F|}_{x=0}\left(t\right)}{ℳ} \end{array}$$

as the end point acceleration. Once the acceleration distribution is known, the corresponding force distribution can be calculated according to (1).

With *F*|_*x*=0_(*t*) being the force difference between the resting (here: at *x* = 0) and the most accelerated (here: at *x* = *l*_*M*_(*t*)) muscle part, and with ${\ddot{x}|}_{x=l_{M}}\left(t\right)$ being the difference in acceleration between both of these muscle parts, we may define

(5) $$\begin{array}{c} \mu_{\text{eff}}\left(t\right)=\frac{{F|}_{x=0}\left(t\right)}{{\ddot{x}|}_{x=l_{M}}\left(t\right)} \end{array}$$

as the “effective mass” of a muscle for which inertia, forces, and kinematics are distributed along its longitudinal axis. According to (4),

(6) $$\begin{array}{c} \mu_{\text{eff}}=\frac{ℳ}{2} \end{array}$$

would be an indicator of accelerations increasing linearly from the fixed to the free muscle end.

## 3. Results

The maximum contraction velocity *v*_max⁡_ is generally a function of of activity *q* [21, 40] and length *l*_CE_ [21, 40–43]: *v*_max⁡_(*l*_CE_, *q*) = *B*_*rel*⁡_(*l*_CE_, *q*)/*A*_*rel*⁡_(*l*_CE_, *q*) · *l*_CE,opt_. Essentially, in our case of a fully active muscle (*q* = 1) always at *l*_*CE*_ ≤ *l*_CE,opt_, this means *v*_max⁡_(*l*_CE_) = *B*_*rel*⁡,0_/*A*_*rel*⁡,0_ · *l*_CE,opt_ · *f*_isom_(*l*_CE_) [21, 40] with *A*_*rel*⁡,0_ = *A*_*rel*⁡_(*l*_CE_ = *l*_CE,opt_, *q* = 1), *B*_*rel*⁡,0_ = *B*_*rel*⁡_(*l*_CE_ = *l*_CE,opt_, *q* = 1) [21]. However, the time periods analysed here are short enough for the CEs not to shorten by more than 0.25 · *l*_CE,opt_. Thus, *v*_max⁡_ is well approximated by its absolute maximum value *v*_max⁡,0_ = *B*_*rel*⁡,0_/*A*_*rel*⁡,0_ · *l*_CE,opt_ (see Section 3.3 below: *v*_max⁡_ > 0.95 · *v*_max⁡,0_).

### 3.1. Smaller Muscle

**Figure 2:**
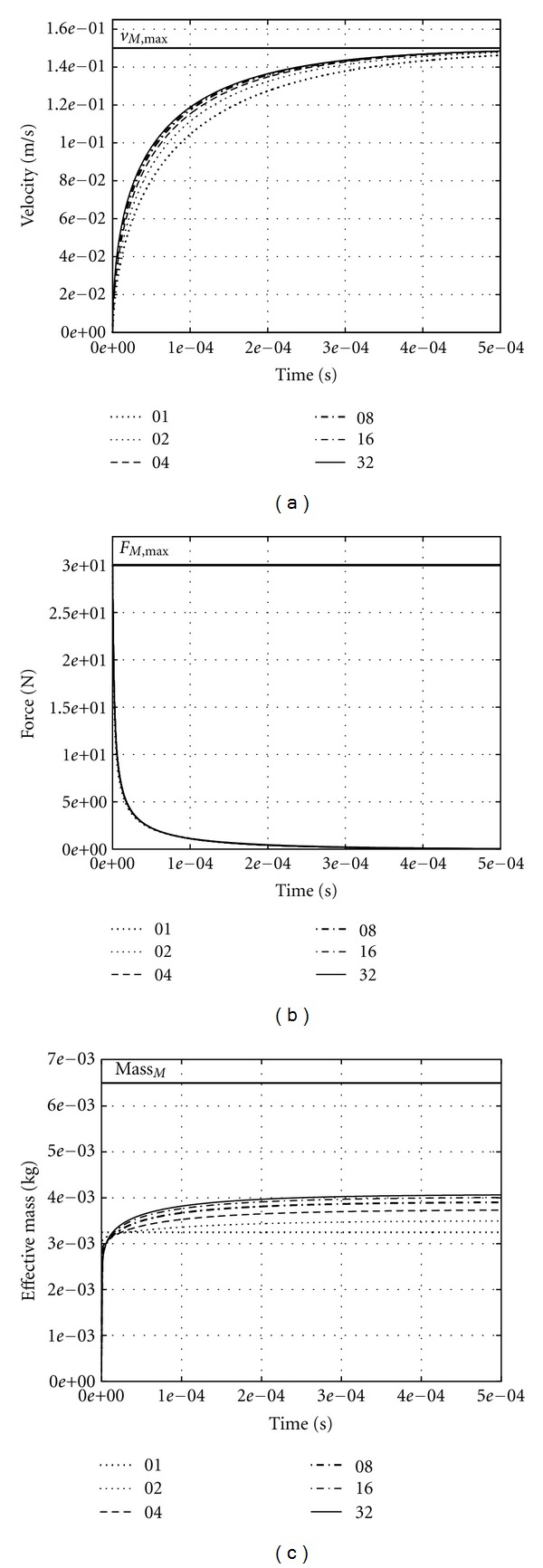
Computer simulation of accelerated contractions of six muscle models with the same overall mass (6.5 g), maximum isometric force (30 N), optimal length (0.015 m), and maximum contraction velocity (0.15 m/s). The muscles were fixed at one end and always fully active (*q* = 1). Models differ just with respect to the number of accelerated discrete point masses approximating a continuous distribution of muscle mass. The point masses were connected by an equal number *I*_*M*_ (see insets in Figure 2) of contractile elements. Their respective optimal lengths *l*_CE,opt,*i*_ were chosen equal to the optimal muscle length *l*_*M*,opt_ divided by *I*_*M*_. The graph depicts the velocity of the point mass at the free end (a), the force at the fixed end (b), and the effective mass (c), that is, the ratio of force at the fixed end and acceleration of the point mass at the free end, versus time. The effective mass to be expected for an exactly linear acceleration distribution along the muscle would be half of the muscle mass (Mass_*M*_ = *ℳ*; (6): *μ*_eff_ = *ℳ*/2 = 3.25 g). The analytic solution for one CE accelerating one point mass predicts (see Appendix A) a typical time of 3.6 · 10^−4^ s for this muscle to approach *v*_max⁡_.

In Figure 2, the simulation results of a contraction with one end fixed are shown for the smaller muscle (*ℳ* = 6.5 g, *F*_max⁡_ = 30 N, *l*_*M*,opt_ = 0.015 m). After about 0.5 ms, the PM at the free end of the muscle (and, thus, the whole muscle) has reached *v*_max⁡_ (Figure 2(a)). Then, the muscle has contracted by about 0.01 · *l*_*M*,opt_. Modelling just one CE (and, thus, one PM free to move: *I*_*M*_ = 1) already approximates the rise in contraction velocity of a continuous mass distribution. An analytic solution for the force step response of one CE accelerating the overall muscle mass (see Appendix A) confirms our numerical results. The typical time constant for this muscle, when approaching *v*_max⁡_, is 3.6 · 10^−4^ s. The initial response at *v* = 0 is faster by two orders of magnitude.

The mass distribution is well approximated by modelling few tens of PMs as the difference between the time courses of the *I*_*M*_ = 16 and *I*_*M*_ = 32 models is not discernible any more in Figure 2. Due to the hyperbolic force-velocity relation the force exerted at the fixed end drops even sharper (Figure 2(b)) than velocity rises. The *I*_*M*_ = 1 model is enough to predict the dynamics of this force decay. The ratio between the fixed end force and the acceleration of the PM at the free end (i.e., their differences between both ends, resp.), which defines the effective mass *μ*_eff_ (5), reaches its stationary value after about the very time in which the force has dropped to almost zero (0.2 ms: Figure 2(c)).

Not surprisingly, we always get exactly *μ*_eff_ = *ℳ*/2 (6) in case of *I*_*M*_ = 1 because (3) is consistently fulfilled throughout. In case of *I*_*M*_ > 1, the initial value is *μ*_eff_ = *ℳ*/*I*_*M*_ (i.e., lim⁡_*I*_*M*_→*∞*_(*μ*_eff_) = 0) because maximum isometric force acts initially on both sides of each PM, except for the very PM at the free end. With increasing *I*_*M*_ the stationary effective mass value converges to a little more than *μ*_eff_ = *ℳ*/2. Note that *μ*_eff_ = *ℳ*/2 is predicted in (4) exactly (6), if the PM accelerations increase linearly with the distance *x* from the fixed to the free end (3). A stationary effective mass nearby *μ*_eff_ = *ℳ*/2 indicates such an approximately linear acceleration (and, thus, force) distribution already after about 0.2 ms. Subsequently, the muscle still accelerates (see time course of the velocity), albeit slightly and at low forces.

### 3.2. Scaling

Here, we condense the scaling characteristics of the muscle model, going without explicit plots. In our one-dimensional model, which assumes a homogeneous mass distribution along the muscle, the parameters *A*_*rel*⁡_, *B*_*rel*⁡_ of the hyperbolic force-velocity relation fully determine the dimensionless time courses of forces and kinematic variables (length, velocity, acceleration). As a consequence thereof, the effective mass scales in proportion to force and time, respectively, and reciprocally to length (5). When varying solely one single muscle parameter, we find the following strictly linear scaling:

- forces just scale with *F*_max⁡_;
- kinematic variables just scale with *l*_*M*,opt_;
- time (i.e., length of time responses) scales with *ℳ*/*F*_max⁡_ and with *l*_*M*,opt_ (see also Appendix A).

As we see in the next paragraph, time does still scale approximately with fixed *ℳ* · *l*_*M*,opt_/*F*_max⁡_; however, the dimensionless time courses of all variables are biased. Note that we express scaling with the maximum isometric force *F*_max⁡_ instead of the current isometric force *F*_CE,isom_ because (i) *F*_max⁡_ is a measure of cross-sectional area in muscles (maximum stress is nearly a constant across muscle dimensions), and (ii) our simulations are performed with a fully activated muscle around the optimal length.

### 3.3. Bigger Muscle

**Figure 3:**
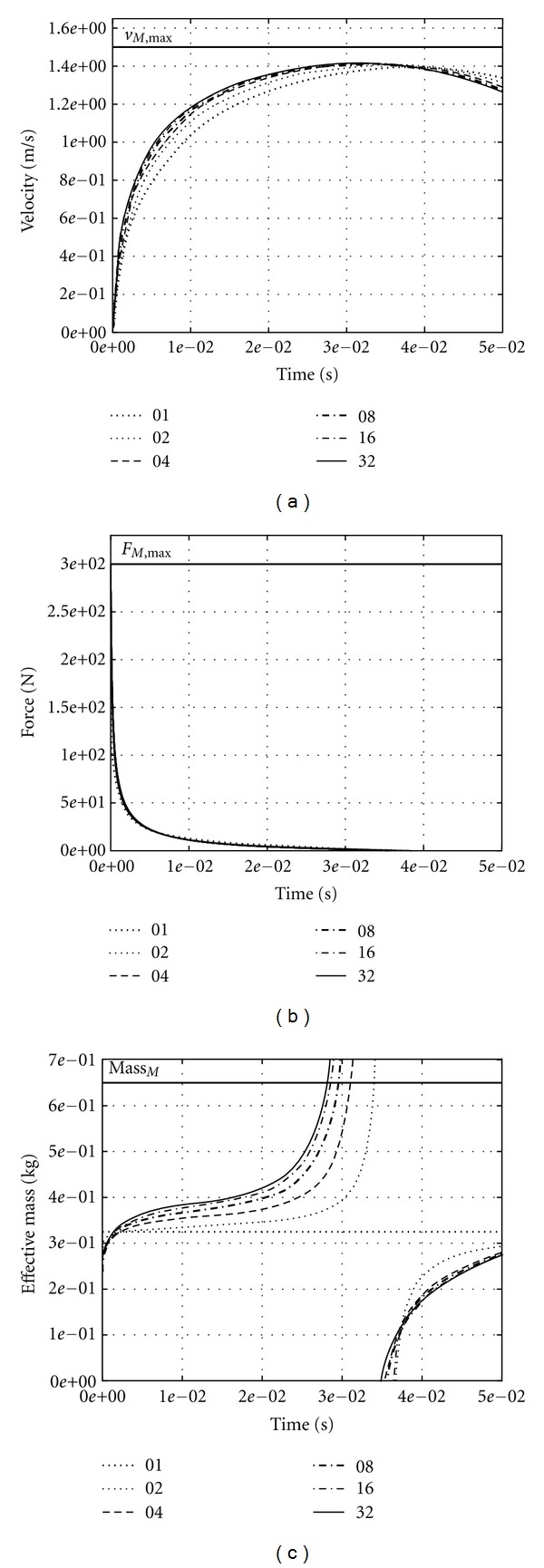
Computer simulation of accelerated contractions of six muscle models with the same overall mass (650 g), maximum isometric force (300 N), optimal length (0.15 m), and maximum contraction velocity (1.5 m/s). The muscles were fixed at one end and always fully active (*q* = 1). Models differ just with respect to the number of accelerated discrete point masses approximating a continuous distribution of muscle mass. The point masses were connected by an equal number *I*_*M*_ (see insets in Figure 3) of contractile elements. Their respective optimal lengths *l*_CE,opt,*i*_ were chosen equal to the optimal muscle length *l*_*M*,opt_ divided by *I*_*M*_. The graph depicts velocity (a), force (b), and effective mass (c) versus time. The effective mass to be expected for an exactly linear acceleration distribution along the muscle would be half of the muscle mass (Mass_*M*_ = *ℳ*; (6): *μ*_eff_ = *ℳ*/2 = 325 g). The analytic solution for one CE accelerating one point mass predicts (see Appendix A) a typical time of 3.6 · 10^−2^ s for this muscle to approach *v*_max⁡_. Note the hundredfold muscle mass, tenfold maximum isometric force and optimal length, respectively, and hundredfold time scale as compared to the results presented in Figure 2.

The course of the force (Figure 3(b)) in a muscle representing an upscaled version (*ℳ* = 650 g, *F*_max⁡_ = 300 N, *l*_*M*,opt_ = 0.15 m) of the one in Section 3.1 proves that the time scale is hundredfold, as expected due to tenfold *ℳ*/*F*_max⁡_ and ten-fold *l*_*M*,opt_. Yet, the courses of the velocity (Figure 3(a)) and effective mass (Figure 3(c)) also demonstrate that contraction kinematics is biased. The effective mass does not saturate, as in the smaller muscle (Figure 2(c)), before end point acceleration has reached zero. This occurs at about 35 ms for *I*_*M*_ = 1 and at about 30 ms for *I*_*M*_ = 32 (approximating continuous mass distribution *I*_*M*_ → *∞*), leading to a pole in the effective mass (Figure 3(c)). This also means that, due to mass inertia, the force in the (outermost: *i* = *I*_*M*_) CE acting on the PM at the free end vanishes earlier than that of the opposite (*i* = 1) CE at the fixed end. As a consequence, in the instant when the PM at the free end is not accelerated any more (see the pole in effective mass), since the CE force has dropped to zero (thus, the outermost CE has reached *v*_max⁡_), the inner elements (closer to the fixed end) shorten still slightly below *v*_max⁡_. Note that the muscle has contracted by about 0.24 · *l*_*M*,opt_ after 30 ms (compared to the smaller muscle: there, *v*_*M*,max⁡_ is reached already after about 0.5 ms with about 0.01 · *l*_*M*,opt_ change in length; see Section 3.1). That is, at this instant *v*_*M*,max⁡_ has additionally dropped by (but not more than: due to broad ascending branch of force-length relation [21]) about five percent below its absolute maximum value *v*_*M*,max⁡,0_ = *B*_*rel*⁡_/*A*_*rel*⁡_ · *l*_*M*,opt_ due to its length dependency. This latter effect superposes with the fact that the inner elements still contract at submaximal velocity at this instant. As a result, the theoretical absolute maximum *v*_*M*,max⁡,0_ is never reached.

In our model, muscle velocity decreases after that instant of zero force at the free end, occurring at 30 ⋯ 35 ms. The corresponding energy dissipation originates from those CEs that contract beyond their *v*_max⁡,*i*_(*l*_CE,*i*_) (due to kinetic energy of the adjacent PM). In this loading situation, we allowed such low compressive forces (*F*_CE,*i*_ < 0) that would arise from steadily extrapolating the hyperbolic force-velocity relation to *v*_CE,*i*_ > *v*_max⁡,*i*_(*l*_CE,*i*_). Physiologically, some low compressive forces should in fact occur in real muscles as these are always somehow constrained in transverse direction. Whether this steady extrapolation is backed physiologically remains open owing to a lack of literature data. Due to such compressive forces, we find a maximum in the free end's velocity, which is lower than the absolute maximum value *v*_*M*,max⁡,0_ that would be theoretically expected from pure CE properties. Even without compressive forces, the theoretical *v*_*M*,max⁡,0_ would never be reached. This is due to the fact that mere interaction of mass inertia and force-velocity relation delay the increase in contraction velocity long enough for part of the CEs to shorten into the ascending branch of their force-length dependency.

## 4. Discussion

### 4.1. Inertia in Finite Element Models

Besides modelling skeletal muscles using Hill-type models, there exist many other modelling approaches. For this study, Hodgkin-Huxley models aiming to investigate electrophysiological cellular properties [44, 45], structural three-dimensional models using the governing equations of finite elasticity to describe muscle mechanics [46–50] and combinations thereof [51, 52] seem to be of primary interest. Hodgkin-Huxley-like models typically focus only on the electrophysiological properties and hence ignore muscle mass entirely. In the case of structural models, mass is included within the governing equations of finite elasticity through mass density, representing weight forces and inertia terms. Except for [47, 53], inertia terms are typically ignored. In most cases, quasi-static approaches are adopted, which simplify, from a computational point of view, the governing equations of finite elasticity. Ignoring muscle inertia and appealing to quasi-static approaches might be a valid assumption when considering isometric contractions or slow motions. However, as shown here, there exist contraction scenarios in which it is no longer justifiable to ignore muscle inertia in structural models.

### 4.2. Contraction Dynamics of Second Order?

Muscle inertia must be taken into account in specific modes of contraction, particularly in responses to impact loads [54–60]. In such a case, whole muscle contraction dynamics is of second order. In concentric contractions, muscle masses do not seem to play a relevant role because the characteristic of contraction dynamics was originally found to be a force-velocity relation, namely, the hyperbolic Hill relation [1]. The relation definitely depends explicitly on the external load (force), whereas it is explicitly independent of internal muscle inertia. However, force-velocity relations have ever been determined in preparations of isolated small muscles of a few grams rather than in isolated muscles of big mammals. So far, in these preparations of isolated small muscles inertia forces have not been found to bias contraction dynamics, at least not in muscle states at a few milliseconds after release.

In contrast, our simulation results predict that maximum concentric contraction velocity, being one parameter in the Hill relation, cannot be directly measured in whole muscle preparations of big mammals. The other way round, if the hyperbolic force-velocity relation really mapped the concentric muscular contraction dynamics microscopically [61, 62] and macroscopically [1, 63], then a big muscle would never reach the corresponding maximum contraction velocity at zero load (Figure 3(a)). In big muscles, therefore, muscle internal inertia should have to be factored in, even during concentric contractions, when striving to identify the contractile properties only. Consequently, a first-order force-velocity relation would not be expected to fully describe the macroscopic dynamics of larger muscles.

### 4.3. Rapid Responses in Isotonic after Load Experiments: Just Elastic?

Contractions of frog fibre bundles with *ℳ* = 0.06 g, *F*_max⁡_ = 0.3 N, *l*_CE,opt_ = 3.0 cm have been examined in a former study [13]. The muscle force per mass ratio between our small (piglet-like) muscle and a frog fibre bundle, which has a hundredth of our muscle's cross-sectional area, is about the same. Consequently, time characteristics should approximately be equal. The authors [13] stated: “We conclude that the change of velocity follows the change in force very quickly probably in less than 1 msec and certainly in less than 6 msec.” According to our simulations which include mass inertia, *v*_max⁡_ is reached after about 0.5 ms. Thus, the question arises whether the time for levelling off at the new force in isotonic quick-release contractions is really just due to serial (visco-)elasticity rather than a mixture of (visco-)elastic and inertia properties.

In other experiments on single frog fibres [64], again with a comparable force per mass ratio, the measuring device had an internal elasticity (in series with the muscle) with an unloaded eigenfrequency of about 200 Hz, corresponding to a time period of 5 ms. As an “initial elastic response” within about 1 ms can be estimated from their plots on contracting muscle, we would conclude that the muscle stiffness must have been much higher than the device stiffness. Without any device stiffness the usually proposed “initial elastic response” should have occurred even faster than within 1 ms. If this was true, the rapid response to force steps would even more likely be a superposition of (visco-)elastic and inertia effects.

### 4.4. Experimental Implications for Studying Inertia in Muscle Preparations

In case one aims to examine inertia effects in the rapid responses of small muscle preparations (as frog, toad, piglet) experimentally, measuring devices of very low inertia, high stiffness, and at least 10^−6^ s time resolution were indispensable. A force step should be favoured as the experimental protocol, rather than a length or velocity step. This is, because the interpretation of the experimental results of kinematic disturbances depends on assuming unknown material characteristics, whereas a force step is a disturbance provoking the contractile kinematics in a natural cause-effect chain, that is, not interfering with the material characteristics during the (ideally infinitely short) step. As to our knowledge, there has been one modern study [27] fulfilling already three of the experimental requirements: it used a force-step protocol for frog muscles with time resolution of 10^−6^ s. Unfortunately, the resonance frequency of the apparatus was not more than 5 · 10^4^ Hz. Eventually, they applied force steps at a length of about 10^−4^ s. To resolve inertia effects, a setup tuned for step lengths of at least 10^−6^ s would be required (ideally 10^−7^ s), while choosing for a time resolution of at least the step length, accordingly. As an alternative, longer fibres could be used. Additionally, any tendinous material in series to the fibres should be removed as good as possible, and the stiffness of the apparatus should always be clearly higher than that of any remaining tendon material.

### 4.5. Muscle-Inertia Interaction in Nonstationary Conditions

If the hyperbolic force-velocity relation *F*(*v*) is linearised at any operating point, an analytic solution can be derived for the dynamic response of the muscle model (including an accelerated mass) to a step in the external force (see Appendix A). This solution predicts an aperiodic exponential decay of the force step towards a limit velocity. Furthermore, limit velocity and time-constant *τ* of the decay vary along the operating points *F*(*v*). In fact, *τ* is simply the reciprocal of the current slope of the force-velocity hyperbola times the accelerated mass. Therefore, depending on the curvature, the time-constant *τ* of the decay can vary one to two orders of magnitude across all possible contraction states of a muscle. Thus, adaptations to force steps happen quickly for contractions with high force and slow velocity (i.e., fastest around the isometric state), while it happens much slower at low forces and high velocities. In the example calculated in Appendix A (parameters of our smaller muscle from Section 3.1), we find *τ* = 3.0 · 10^−6^ at *v* = 0, and *τ* = 3.6 · 10^−4^ at *v* = −*v*_max⁡_.

This step response is induced by the interaction of inertia and force-velocity relation. In a technical sense, this characteristic represents a low-pass filter with a state-dependent cut-off frequency. Mechanically spoken, the force-velocity relation is equivalent to a damper with a state-dependent damping coefficient. This is in accordance with a recently proposed macroscopic model for the CE [65]. In that model, the CE consists of three simple mechanical elements: an active energy source (AE), a parallel damper (PDE), and a serial element (SE). There, the PDE is assumed to be a viscous damper, with the damping coefficient depending linearly on the CE force (high damping for high CE forces). With this assumption, the model predicts the operating points on the hyperbolic force-velocity relation from the force equilibrium of the three elements. Obviously, the differential equation defining a hyperbola (see ((A.8)) in Appendix A) is mathematically equivalent to a manifold of force equilibria of the CE elements such as proposed in [65]. All in all, it seems the muscle's hyperbolic force-velocity relation can be viewed from at least three perspectives, which are equivalent. (i) It can be considered a linearly force-dependent damping coefficient for external inertia loads. (ii) It performs as a low-pass filter for whole muscle contraction exposed to external steps in force. And (iii) it represents a potential to compensate (homogenise) muscle internal force and velocity distributions.

In first attempts to implement the above-mentioned CE as a mechanical assembly [66, 67], the SE was realised with a linear mechanical spring introducing additional elasticity (in series to possible SEE elasticity representing tendon and aponeurosis). Such a CE-internal elasticity from the SE superposes oscillations to the above-described aperiodic exponential decay. Alternatively, this CE model allows also to detail the assumed SE properties as viscoelastic or purely damping. Each would be expected to reduce any oscillatory behaviour. Both in technical implementations as proposed above and in experimental preparations of physiological muscle, these effects should be as much visible and, thus, verifiable. Such experiments could, therefore, help to further reveal the internal structure of assemblies of active muscle fibres and help to push forward CE models proposed to represent them. Heat measurements on muscles are another means to check for the validity of different CE models with the same force-velocity relation but different internal designs (element properties) [65]. We would expect that heat measurements should also be prone to inertia effects, may they come from muscle internal masses or potential inertia of the measuring device [68].

As a first approximation we can usually consider muscle mass *ℳ* to be scaled geometrically by either changing its cross-sectional area *S* or its length as represented by its optimal fibre length *l*_*M*,opt_. This implies that both mass density *ρ* and maximum stress *σ*_max⁡_ are about invariant parameters of muscles. Thus, in a real muscle its mass is not an independent parameter as in our model, and the rule to scale contraction times in proportion to *ℳ* · *l*_*M*,opt_/*F*_max⁡_ as expressed in Section 3.1 and in ((A.11)) and ((A.12)) can be condensed to ((A.13)). We should be aware of the fact that ((A.11)) and ((A.12)) do apply to any contracting muscle, may it accelerate just its internal mass (as analysed numerically in this paper) or an external mass that is usually much higher than its own mass. In contrast, ((A.13)) applies to the specific case of a muscle contracting just against its internal mass under the above-mentioned approximative assumptions of constant density and maximum stress. Thus, ((A.13)) tells us that the contraction time for a muscle exposed to a step in force, rather than analysed when accelerating an external load, scales in fact with the square of its fibre length. We would, therefore, expect that the effect of not reaching the theoretical maximum contraction velocity *v*_max⁡_ (Figure 3(a)) would occur in any muscle preparation with fibre lengths that exceed a value somewhere between 1.5 cm and 15 cm, regardless of muscle mass. In the end, it seems that it is not the value of the muscle mass itself but the fibre length that scales the response times to force steps (even quadratically, see ((A.13))). The effect occurs because contraction times for the whole muscle increase due to the force gradient decreasing with muscle extension, whereas the outermost muscle portions (released immediately in a force step) are always accelerated to their local *v*_max⁡_ (and already decelerated consecutively due to local compression) much faster on an invariant time scale. For a 15 cm fibre, a muscle is predicted to reach just 93% of *v*_max⁡_.

We dare this preliminary prediction subject to future theoretical analyses explicitly including muscle internal elasticity. Serial elasticity might not really help to further speed whole muscle acceleration up. Parallel elasticity, however, might be effective because it adds accelerative forces to the force-velocity relation. Yet, interaction with damping like the force-velocity relation itself and with the mass distribution in series might reduce its accelerating effect down to a secondary level. Parallel elasticity is also a challenge to experimenters because it usually biases contraction measurements as soon as they are performed above or even at optimal fibre lengths. To make our theoretical analysis of the force-velocity relation contributing to accelerated contractions as comparable as possible with experiments, we chose initial conditions at which parallel elasticity is (almost) negligible, that is, at optimal lengths. As a result, our bigger muscle has already contracted by about 0.24 · *l*_*M*,opt_ in the instant when it starts to decelerate its all over contraction (see first paragraph of Section 3.3). Because our model force-length relation has an unusually broad ascending branch [21] the isometric force for a CE shortened by considerable 24% has only decreased a little to about 95% of its absolute maximum. Therefore, it seems that reaching a contraction velocity of just 0.93 · *v*_max⁡_ is due to a mixture of local compressive forces and the force-length relation in our model simulations. Additional parallel elasticity would only have the potential to compensate for the latter relation. We would, therefore, expect that the phenomenon of not reaching theoretical *v*_max⁡_ in big muscles should also occur when starting at initial lengths above the optimal fibre length.

Finally, we would like to point to another interesting aspect. The propagation velocity of muscle excitation on its surface is about 4 m/s in vertebrate cross-striated muscles [69]. In the smaller muscle, this means that an excitation spike would need about an order of magnitude more time to spread along the complete length of a fibre than to reach *v*_max⁡_ after a maximum force step down to zero: for 1.5 cm fibres we calculated *τ*_*v*=−*v*_max⁡__ = 3.6 · 10^−4^ s, and we find 0.015 m/(4 (m/s)) = 3.8 · 10^−3^ s. As *τ*_*v*=−*v*_max⁡__ scales quadratically with the fibre length (see Appendix A) we calculate, however, *τ*_*v*=−*v*_max⁡__ = 3.6 · 10^−2^ s in the bigger muscle with a tenfold fibre length. In 15 cm fibres the estimated propagation time of 0.15 m/(4 (m/s)) = 3.8 · 10^−2^ s from one end to the other would, thus, already slightly exceed *τ*_*v*=−*v*_max⁡__. Accordingly, when thinking of the muscle response to a twitch as a response to an internal step in force, the propagation velocity, rather than inertia effects, may in fact limit the twitch time for a rise in force up to about 7 cm fibre length (assumed the only end-plate is located in the middle of the fibre). Yet, even in shorter fibres it may be that inertia, rather than excitation propagation, could limit twitch response times because *τ*_*v*=−*v*_max⁡__ increases linearly with muscle stress (see ((A.13))), and twitch stress should be clearly lower than maximum stress during a fused tetanus. Additionally, any twitch time limitation by excitation propagation would be brought nearer to inertia limitation if a couple of end-plates were distributed along a fibre. All in all, excitation propagation contributing to the electromechanical delay restricts response times when a rapid rise to a higher force level, that is, to an increased activity is required [39]. In contrast, inertia effects, rather than excitation propagation, should be a time limiting factor in physiological loading situations when rapid changes in external forces occur at some given activity level at which Ca^2+^ is sufficiently available anyway along the whole fibre.

## 5. Conclusions

We expect that a significant progress in the time resolution of muscle experiments and their sensitivity with respect to measured mechanical variables and heat should allow to verify the predictions made by such models of the contractile muscular machinery that include the muscle mass distribution. The vast majority of current models has neglected muscle inertia and is, thus, not appropriate to understand high-frequency oscillations that have already been seen during some experiments [21, 56, 59, 60, 68, 70–74], let alone even potentially higher frequencies of muscle internal mass repositioning [24–26, 31]. The time scales, on which highly dynamic responses occur when muscle fibre contractions are disturbed by rapid (ideally infinitely steep) force steps, are very likely not to be explainable without muscle inertia. Such responses are eventually expected to depend on three basic mechanical properties: elasticity, viscosity or damping (leading to a release in heat), and inertia. In the model as presented here, we neglected explicit modelling of elasticity (and, therefore, oscillations), but we rather concentrated on the prediction of the time scale on which (exponentially damped) decays of disturbances would occur in case just the well-known hyperbolic Hill relation between force and velocity of active muscle fibres was present. Of course, these time scales would be expected to be also depending on fibre internal elasticity. This is even more the case, because the Hill relation itself has been predicted to possibly emerge from serial (visco-)elasticity that interacts with damping properties [65–67]. Therefore, to explain the superposition of (i) time constants for (exponentially damped) decays of disturbances, (ii) oscillations from the interaction of elasticity and muscle plus external inertia, and (iii) current heat production, thus, to understand muscle design by examining its mechanical and thermodynamical coupling with the environment, more focus on muscle inertia as part of muscle models seems to be indicated. In big muscles, even the asymptotic performance when approaching high velocities at low loads might depend on muscle inertia.

## Appendix

### A. Analytic Solution for a Simplified Acceleration Scenario after a Force Step

To provide a check for correctness of our numerical results, we provide an analytic solution of (2), that is, for the equation of motion of the muscle model's centre of mass (coordinate: *x*_COM_). It describes the corresponding acceleration scenario following a force step, in which the complete muscle mass *ℳ*, located at *x*_COM_, is driven by a Hill-type force element (CE) of length *l* fixed to it (and to the world somewhere at *x* < *x*_COM_), which produces the contractile force

(A.1) $$\begin{array}{c} F_{\mathrm{lin}}\left(v\right)=F_{0}+\frac{F_{0}+A}{B}\cdot v, \end{array}$$

depending on contraction velocity $v=\dot{l}={\dot{x}}_{\text{COM}}$ (negative for concentric contraction). The symbols *A* = *A*_*rel*⁡_ · *F*_0_ (asymptote), *B* = *B*_*rel*⁡_ · *l*_opt_ (asymptote), and *F*_0_ (current isometric force) are the parameters (all positive) of the nonlinear (hyperbolic) Hill relation *F*(*v*) = (*B* · *F*_0_ + *A* · *v*)/(*B* − *v*) (see also (A.7) below). The symbol *l*_opt_ is the optimal value of the CE length *l* on which *F*_0_ depends (as on muscle activity *q*, compare Section 2: *F*_0_ = *F*_0_(*l*, *q*) = *F*_CE,isom,*i*_(*l*_CE,*i*_, *q*_*i*_) for the muscle represented by just one CE with *i* = *I*_*M*_ = 1). For *A*_*rel*⁡_ and *B*_*rel*⁡_ definitions we also refer to Section 2, for their modelling and use see, for example, [21].

Note that we start our analysis with considering just the linearisation *F*_*lin*⁡_(*v*) of *F*(*v*) around the isometric point *v* = 0, *F*(*v* = 0) = *F*_0_ as represented by the term in parentheses on the right hand side of (A.1). The simplified (linearised) force-velocity relation of an active muscle after (A.1) transforms (2) into the linear inhomogeneous differential equation:

(A.2) $$\begin{array}{c} \dot{v}=\frac{-F_{\mathrm{lin}}\left(v\right)}{ℳ}=-\frac{F_{0}}{ℳ}-\frac{F_{0}+A}{ℳ\cdot B}\cdot v. \end{array}$$

The negative sign in −*F*_*lin*⁡_(*v*) is due to making force and velocity (and acceleration) directions consistent to each other: $\dot{v}\left(v=0\right)=-F_{0}/M<0$ and $\dot{v}\left(v=-\left(B\cdot F_{0}\right)/\left(F_{0}+A\right)\right)=0$. The general solution *v*(*t*) = *v*_hom_(*t*) + *v*_inhom_(*t*) of (A.2) is the sum of the solution of the corresponding homogeneous equation (neglecting −*F*_0_/*ℳ* in (A.2))

(A.3) $$\begin{array}{c} v_{\text{hom}}\left(t\right)=e^{-\gamma \cdot t}, \end{array}$$

with the exponent

(A.4) $$\begin{array}{c} \gamma =\frac{F_{0}+A}{ℳ\cdot B}, \end{array}$$

that is, the slope of *F*_*lin*⁡_(*v*) (the damping coefficient *D* = ∂*F*/∂*v* = (*F*_0_ + *A*)/*B*; see (A.1)) divided by the mass *ℳ* and the particular solution

(A.5) $$\begin{array}{c} v_{\text{inhom}}\left(t\right)=C_{0}+C_{1}\cdot e^{-\gamma \cdot t}. \end{array}$$

Requiring (A.5) to fulfil (A.2) means *C*_0_ = −(1/*γ*) · (*F*_0_/*ℳ*), and requiring the specific initial condition *v*(*t* = 0) = 0 fixes *C*_1_ = −1 − *C*_0_. Thus, after all we find

(A.6) $$\begin{array}{c} v\left(t\right)=v_{\text{hom}}\left(t\right)+v_{\text{inhom}}\left(t\right)=\frac{1}{\gamma }\cdot \frac{F_{0}}{ℳ}\cdot \left(e^{-\gamma \cdot t}-1\right). \end{array}$$

As a response to a force step (which is from *F*_0_ to zero in our simulation, starting with *v* = 0), the linearisation *F*_*lin*⁡_(*v*) (A.1) of the hyperbolic Hill relation *F*(*v*) at *v* = 0 would predict that the velocity of the muscle's centre of mass approaches its limit value *v*_end_ = *v*_end,*v*=0_ = lim⁡_*t*→*∞*_(*v*(*t*)) = −(*F*_0_/(*F*_0_ + *A*)) · *B* = −*B*/(1 + *A*_*rel*_) exponentially with the typical time constant *τ* = 1/*γ*. Now, imagine to substitute the local linearisation of the complete nonlinear (hyperbolic) Hill relation

(A.7) $$\begin{array}{c} F\left(v\right)=A\cdot \frac{v_{\max}+v}{B-v}, \end{array}$$

which fulfils exactly (compare (A.1))

(A.8) $$\begin{array}{c} F\left(v\right)=\frac{\partial F\left(v\right)}{\partial v}\left(B-v\right)-A, \end{array}$$

instead of *F*_*lin*⁡_(*v*) at any velocity value *v* into (A.2). The absolute value of the limit velocity *v*_end_ would then simply increase up to *v*_end,*v*=−*v*_max⁡__ = −*v*_max⁡_ = −(*F*_0_/*A*) · *B* = −*B*/*A*_*rel*⁡_ with decreasing after-step force *F*_after_: *v*_end_ = *v*(*F* = *F*_after_) where *v*(*F*) is the inverse function of *F*(*v*). The exponential response would not change in principle, but the typical period *τ* = 1/*γ* would be modified numerically. Equations (A.4) and (A.8) tell us that

(A.9) $$\begin{array}{c} \gamma =\frac{1}{ℳ}\cdot \frac{\partial F}{\partial v} \end{array}$$

is the (current) slope ∂*F*/∂*v* ( = (*F*_0_ + *A*)/*B* at *v* = 0) of the hyperbolic force-velocity relation *F*(*v*), divided by the mass *ℳ*. The slope changes from ∂*F*/∂*v* = (*F*_0_/*v*_max⁡_) · ((1 + *A*_*rel*⁡_)/*A*_*rel*⁡_) at *v* = 0 to ∂*F*/∂*v* = (*F*_0_/*v*_max⁡_) · (*A*_*rel*⁡_/(1 + *A*_*rel*⁡_)) at *v* = −*v*_max⁡_, thus, both the slope ∂*F*/∂*v* and *γ* decrease by the same factor

(A.10) $$\begin{array}{c} r_{\text{slope}}={\left(\frac{A_{\mathrm{rel}}}{1+A_{\mathrm{rel}}}\right)}^{2} \end{array}$$

from *v* = 0 to *v* = −*v*_max⁡_.

Expressing (A.4) in terms of the normalised Hill parameters *A*_*rel*⁡_ = *A*/*F*_0_, *B*_*rel*⁡_ = *B*/*l*_opt_ provides *τ*_*v*_ around *v* = 0, that is, the time constant for an exponential decay from the isometric force *F*_0_ to a new force value *F* = *F*_after_ < *F*_0_ still nearby *F*_0_:

(A.11) τv=0=1γv=0=ℳ·Brel⁡·loptF0+Arel⁡·F0=ℳ·loptF0·Brel⁡1+Arel⁡=ℳ·loptF0·Brel⁡Arel⁡·Arel⁡1+Arel⁡.

Choosing *F*_0_ = *F*_max⁡_ (fully activated muscle at optimal length) and the specific muscle parameter values for our muscle(s) (Section 2: *A*_*rel*⁡_ = 0.1, *B*_*rel*⁡_ = 1.0 (1/s) [21]) we find *τ*_*v*=0_ = 3.0 · 10^−6^ s. For a vanishing after-step force *F* = *F*_after_ = 0 (as in our simulations in this study), the muscle velocity *v* approaches −*v*_max⁡_ where the typical period *τ*_*v*_ is accordingly increased by the factor (1/*r*_slope_) ≈ 100 ((A.10) with *A*_*rel*⁡_ = 0.1):

(A.12) $$\begin{array}{c} \tau_{v=-v_{\max}}=\frac{\tau_{v=0}}{r_{\text{slope}}}=\frac{ℳ\cdot l_{\text{opt}}}{F_{0}}\cdot \frac{B_{\mathrm{rel}}}{A_{\mathrm{rel}}}\cdot \frac{1+A_{\mathrm{rel}}}{A_{\mathrm{rel}}}. \end{array}$$

For our muscle example with *F*_0_ = *F*_max⁡_ we, thus, find *τ*_*v*=−*v*_max⁡__ = 3.6 · 10^−4^ s. The equality *B*_*rel*⁡_/*A*_*rel*⁡_ = *v*_max⁡_/*l*_opt_ may be helpful as more illustrative.

Because the mass *ℳ* is the product of density *ρ* (≈1 g/cm^3^) and volume *V*, the latter scales approximately with the optimal length *l*_opt_ and with the muscle cross-sectional area *S* of the active muscle fibre assembly (*ℳ* = *ρ* · *V* ≈ *ρ* · *c*_*V*_ · *S* · *l*_opt_), and because *F*_0_ can be formulated as scaling with *F*_max⁡_ (see Section 2), the latter being approximately proportional to the product of *S* and the maximum stress *σ*_max⁡_ (≈20 N/cm^2^ across muscles: *F*_0_ = *c*_*F*_ · *F*_max⁡_ ≈ *c*_*F*_ · *σ*_max⁡_ · *S*), we find ((A.11) and (A.12)) that *τ*_*v*_ should scale quadratically with *l*_opt_:

(A.13) $$\begin{array}{c} \frac{ℳ\cdot l_{\text{opt}}}{F_{0}}\approx \frac{c_{V}\cdot \rho }{c_{F}\cdot \sigma_{\max}}\cdot l_{\text{opt}}^{2}. \end{array}$$

Accordingly, we would predict that the exponential time constant for a muscle contracting against its own inertia after externally determined force steps does first and foremost depend on its fibre length rather than on its own mass. If the muscle contracts against an external mass *ℳ* (usually clearly higher than its own) and if there is no further force like, for example, gravity the more general equation (A.12) always applies because the mass is accelerated exponentially until *F*(*v*) has vanished at *v* = −*v*_max⁡_. If there is a further force 0 ≤ *F*_ext_ ≤ *F*_0_ counteracting *F*(*v*), the dynamics is described by an accordingly modified differential equation. Then, (A.11) and (A.12) may still be helpful for roughly estimating (the order of magnitude of) the time that passes by until steady state velocity *v*_max⁡_ ≤ *v* ≤ 0 at *F*(*v*) = *F*_ext_ is reached, namely, some value corresponding to the *F* = *F*_after_ = *F*_ext_ case in between the extremes of (A.11) and (A.12).
